# Supplementary material for: Possible Involvement of Ghost Introgressions in the Striking Diversity of Vomeronasal Type 1 Receptor Genes in East African Cichlids
Source: Ecol Evol. 2025 May 21;15(5):e71467. doi: 10.1002/ece3.71467 (PMC12095850; doi:10.1002/ece3.71467)
Supplement: Supplementary file 1 — Appendix S1. [file ECE3-15-e71467-s002.docx]

Possible involvement of ghost introgressions in the striking diversity of Vomeronasal type 1 receptor genes in East African cichlids

Shunsuke Taki^1^, Zicong Zhang^2^, Mitsuto Aibara^1^, Tatsuki Nagasawa^1^, and Masato Nikaido^1*^

**Tables and Figures**


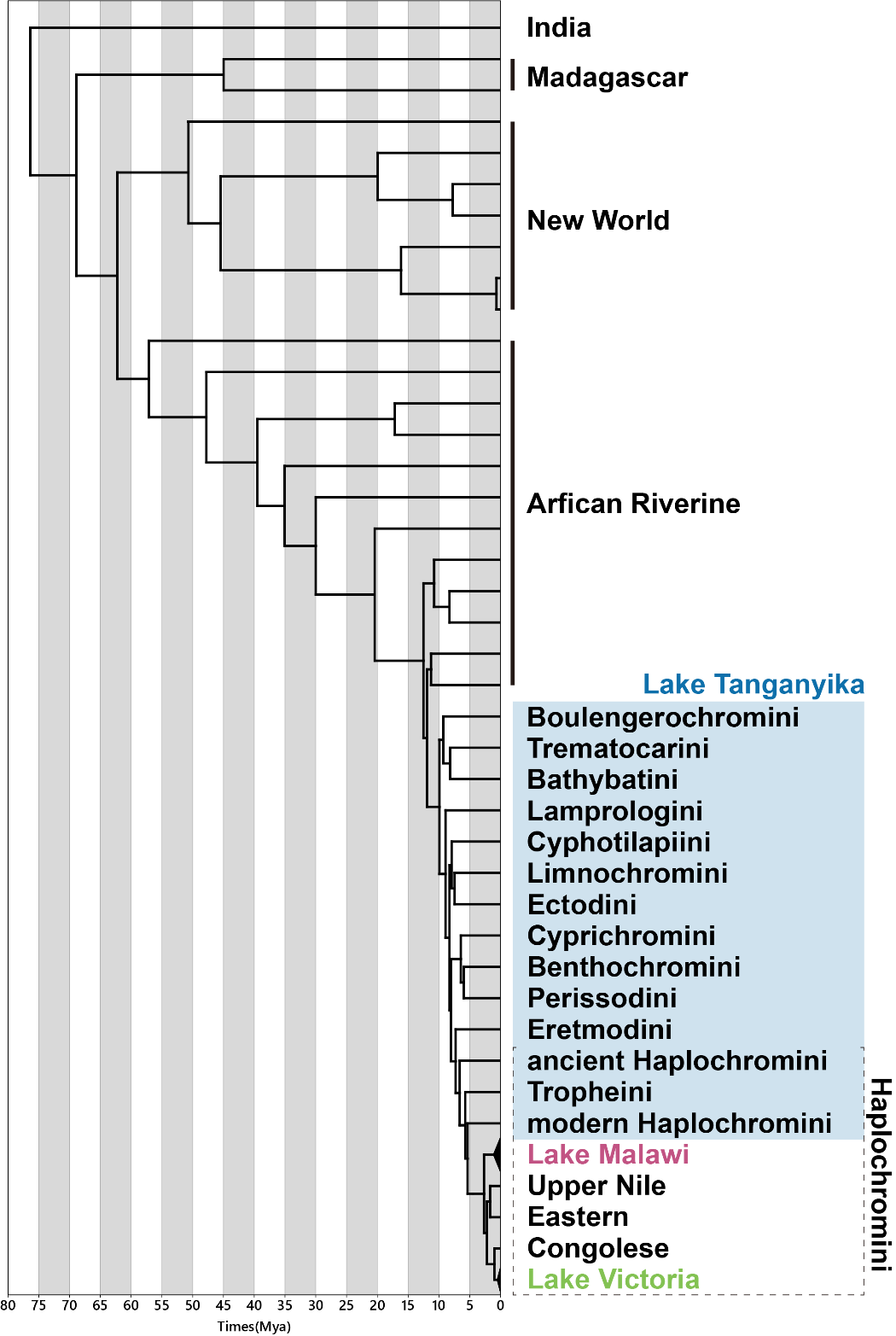


Figure S1 Lineage names in this study are defined with reference to previous studies (Matschiner et al., 2020; Meier et al., 2017; Nakamura et al., 2023; Ronco et al., 2021). Haplochromini species that diverged before Tropheini are defined as "ancient Haplochromini." Divergence times are expressed in millions of years (Mya).


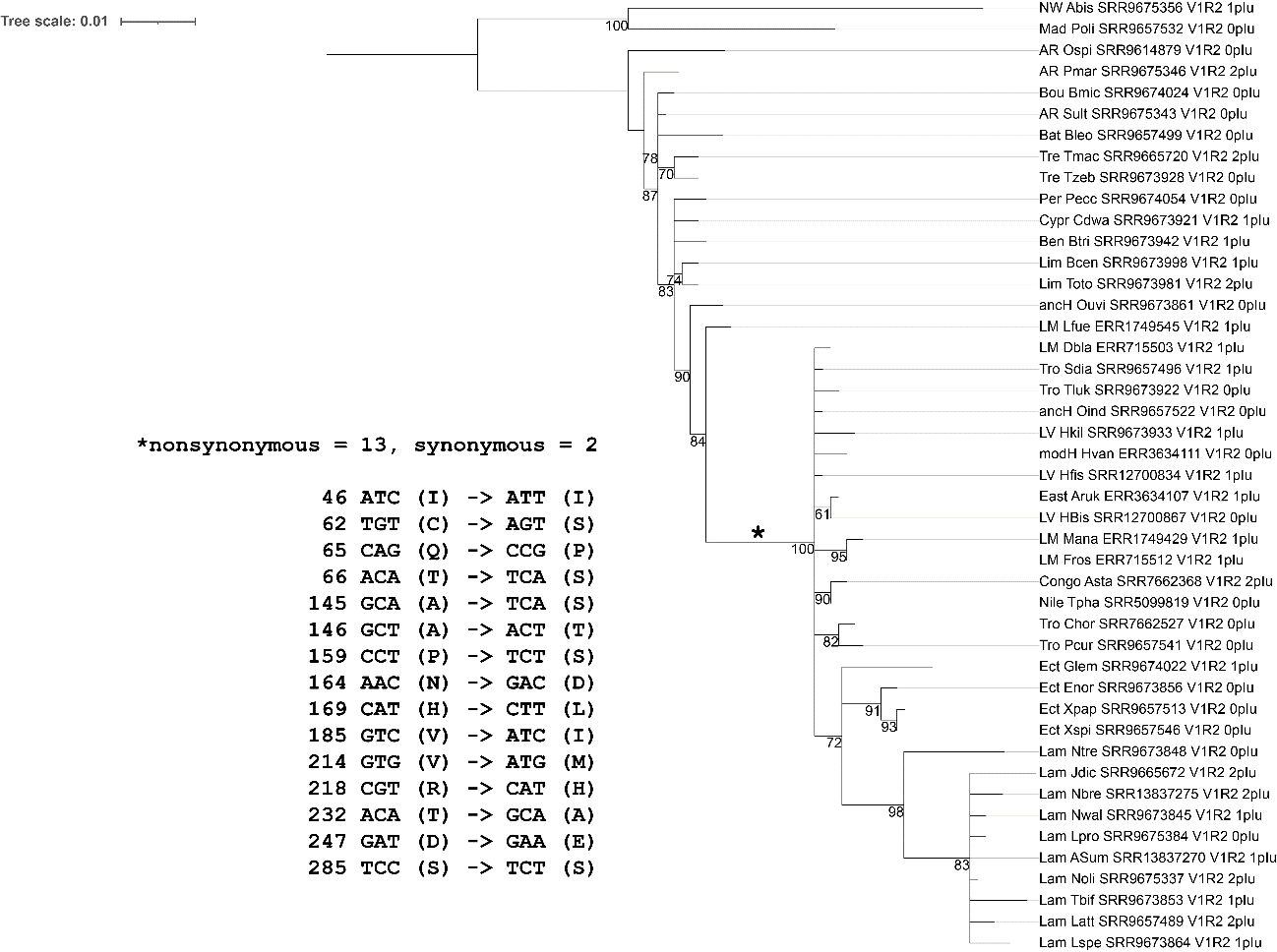


Figure S2 Phylogenetic tree and sequence alignment of the V1R2 gene in cichlids (Dataset 1). The phylogenetic tree displays bootstrap values only for nodes with support values of ≥60. The left panel shows nonsynonymous and synonymous substitution sites at positions marked with an asterisk.


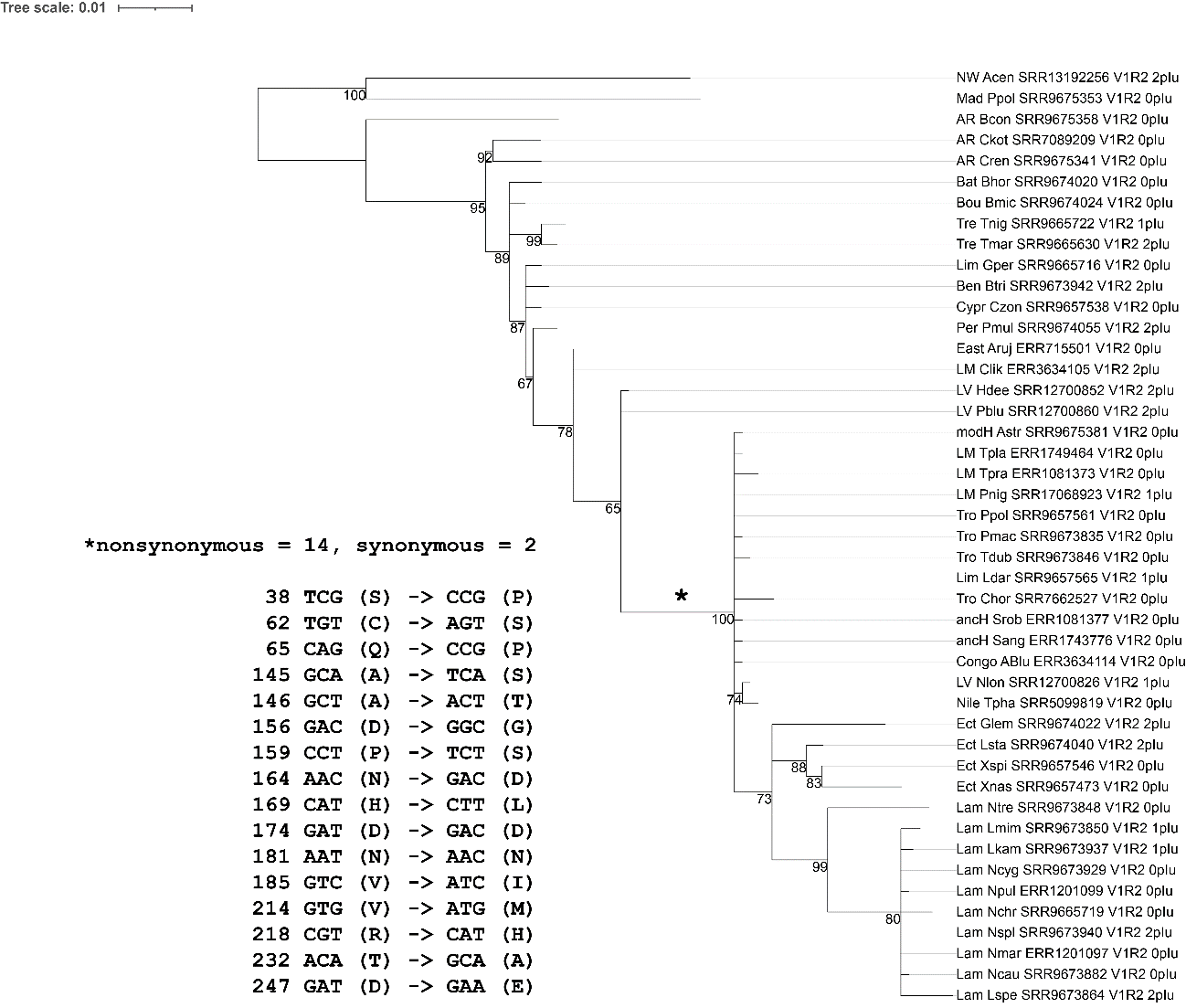


Figure S3 Phylogenetic tree and sequence alignment of the V1R2 gene in cichlids (Dataset 2). The phylogenetic tree displays bootstrap values only for nodes with support values of ≥60. The left panel shows nonsynonymous and synonymous substitution sites at positions marked with an asterisk.


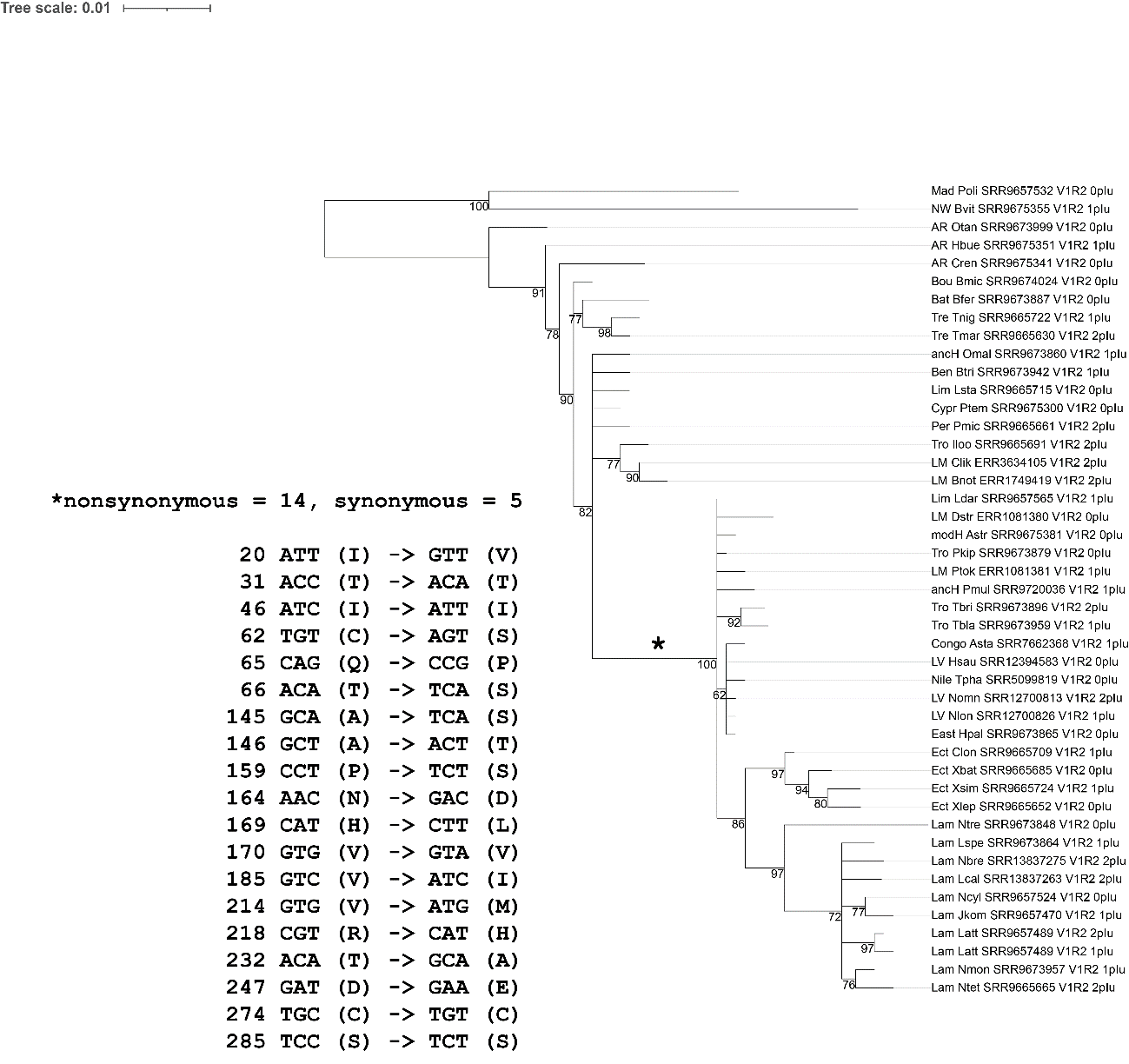


Figure S4 Phylogenetic tree and sequence alignment of the V1R2 gene in cichlids (Dataset 3). The phylogenetic tree displays bootstrap values only for nodes with support values of ≥60. The left panel shows nonsynonymous and synonymous substitution sites at positions marked with an asterisk.


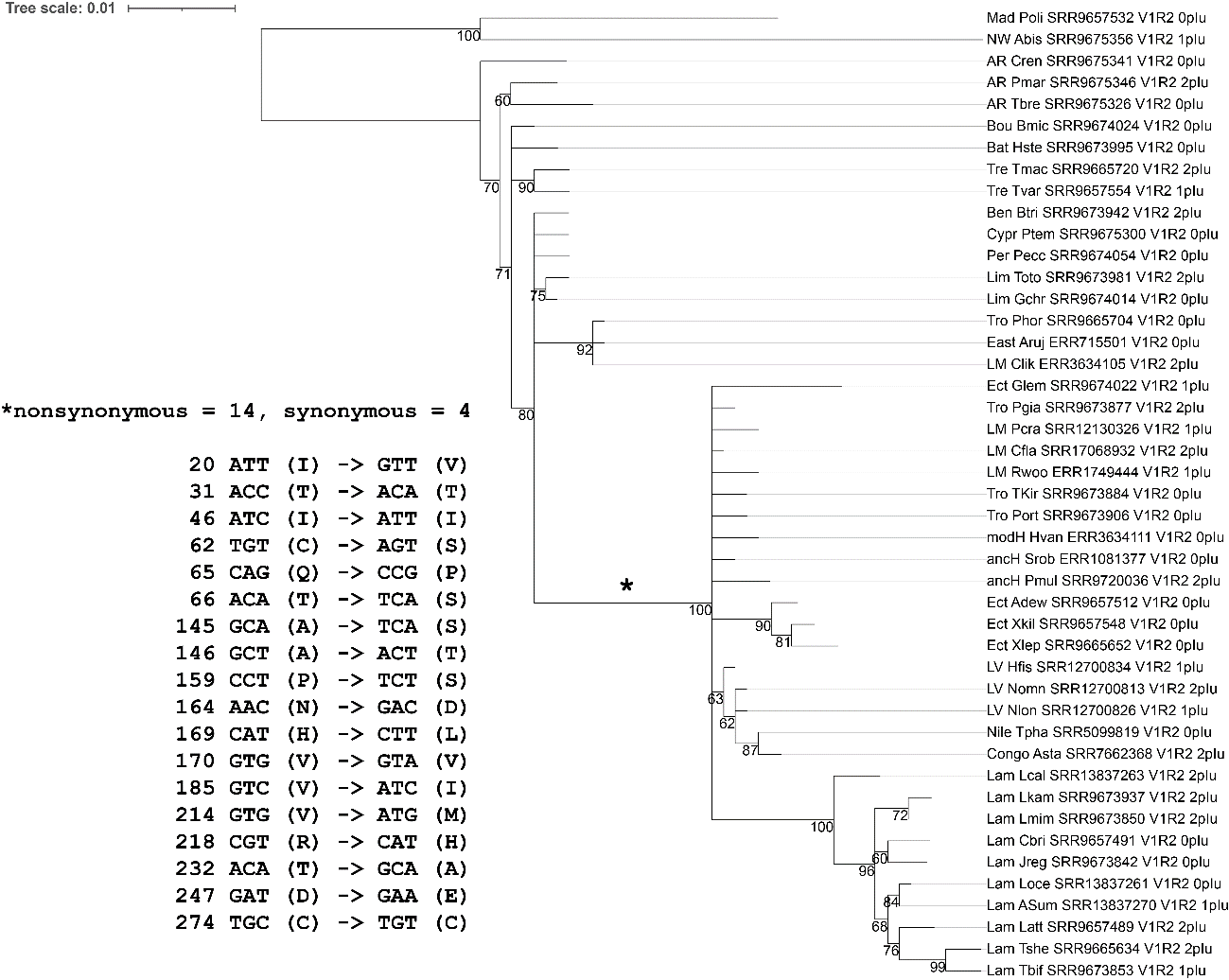


Figure S5 Phylogenetic tree and sequence alignment of the V1R2 gene in cichlids (Dataset 4). The phylogenetic tree displays bootstrap values only for nodes with support values of ≥60. The left panel shows nonsynonymous and synonymous substitution sites at positions marked with an asterisk.


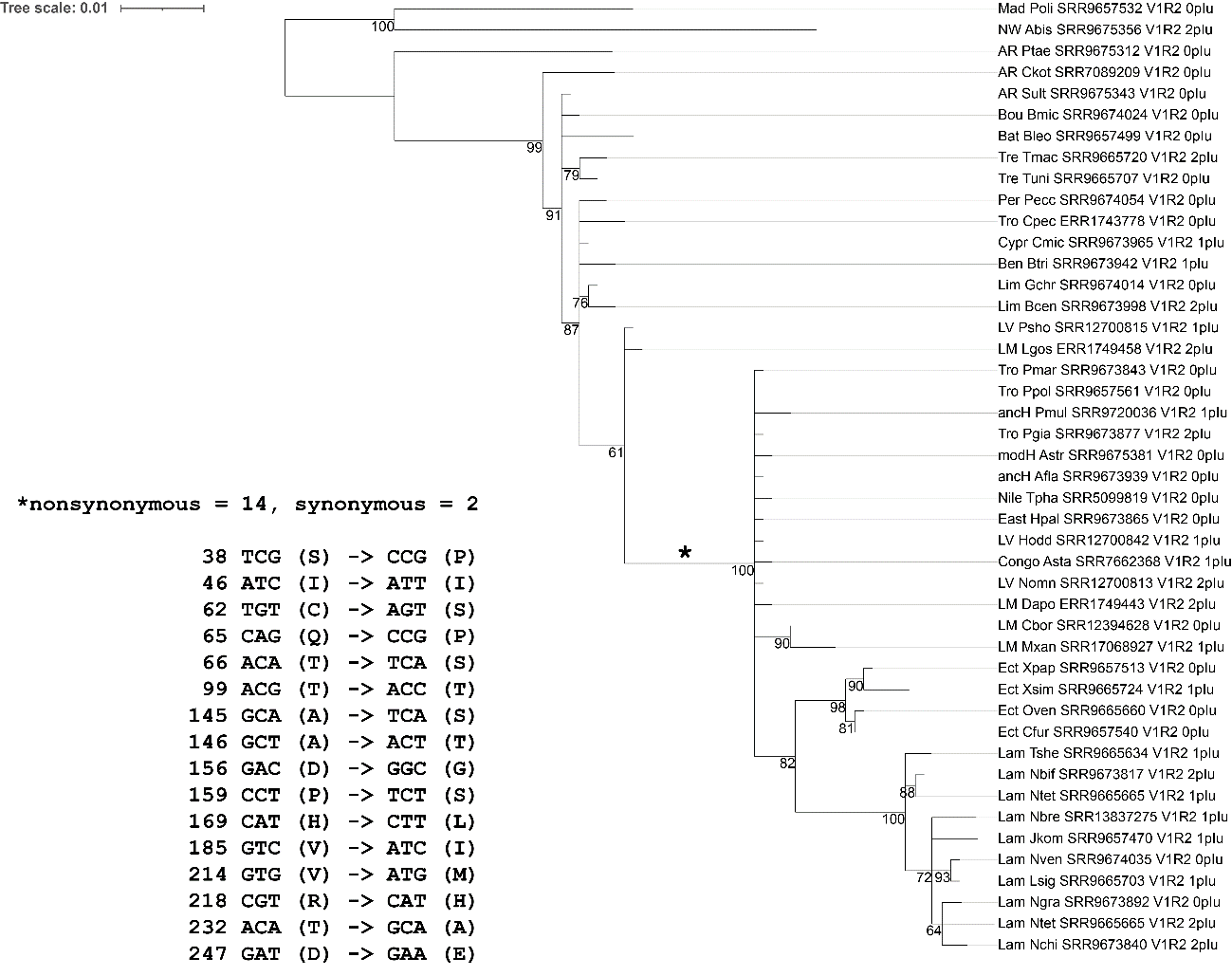


Figure S6 Phylogenetic tree and sequence alignment of the V1R2 gene in cichlids (Dataset 5). The phylogenetic tree displays bootstrap values only for nodes with support values of ≥60. The left panel shows nonsynonymous and synonymous substitution sites at positions marked with an asterisk.


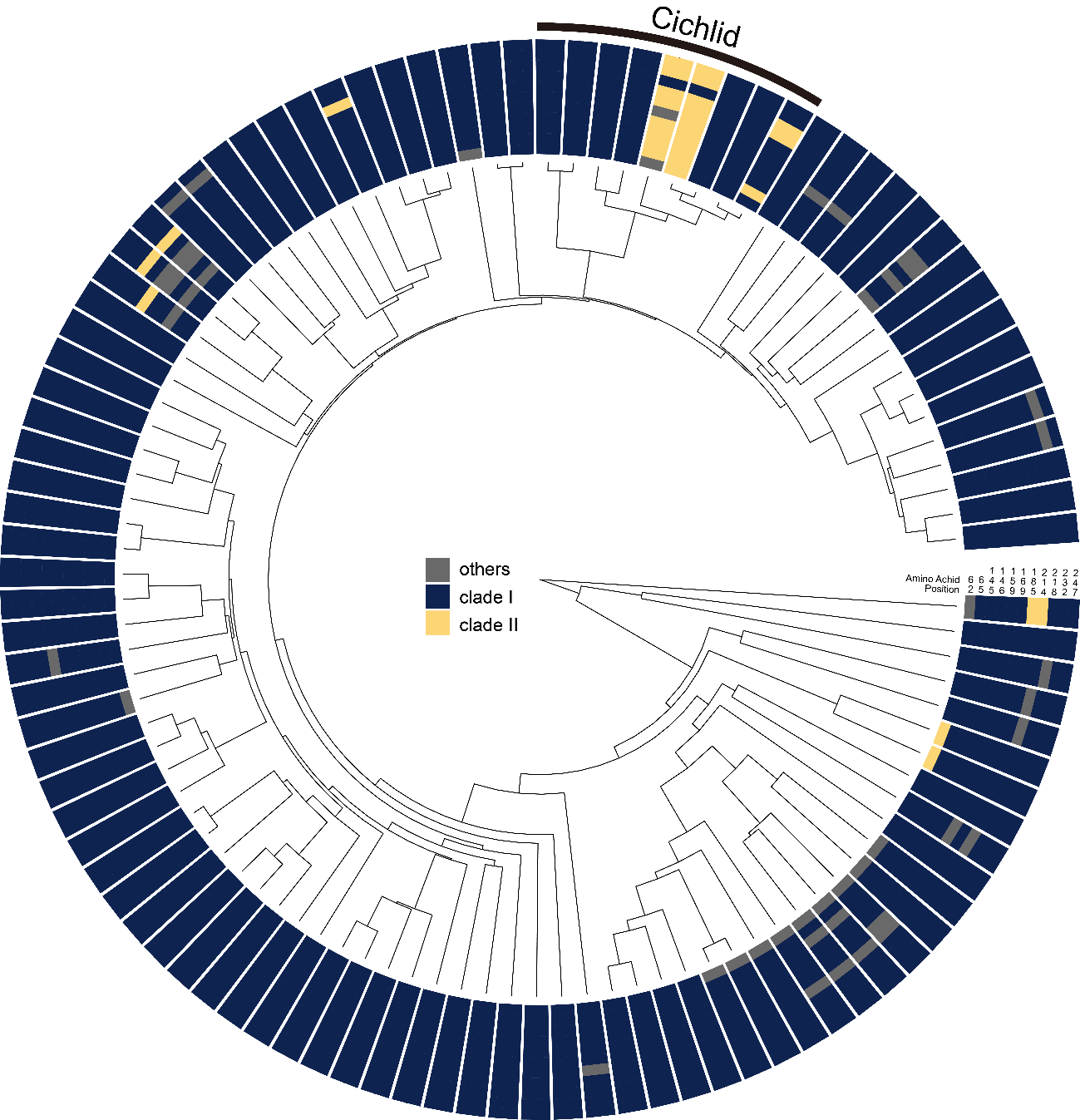


Figure S7 V1R2 alignment for 102 ray-finned fish species. The alignment displays sites corresponding to the two alleles (Clades I and II). Sites matching Clade I are highlighted in dark blue, those matching Clade II are highlighted in yellow, and all other sites are shown in gray.


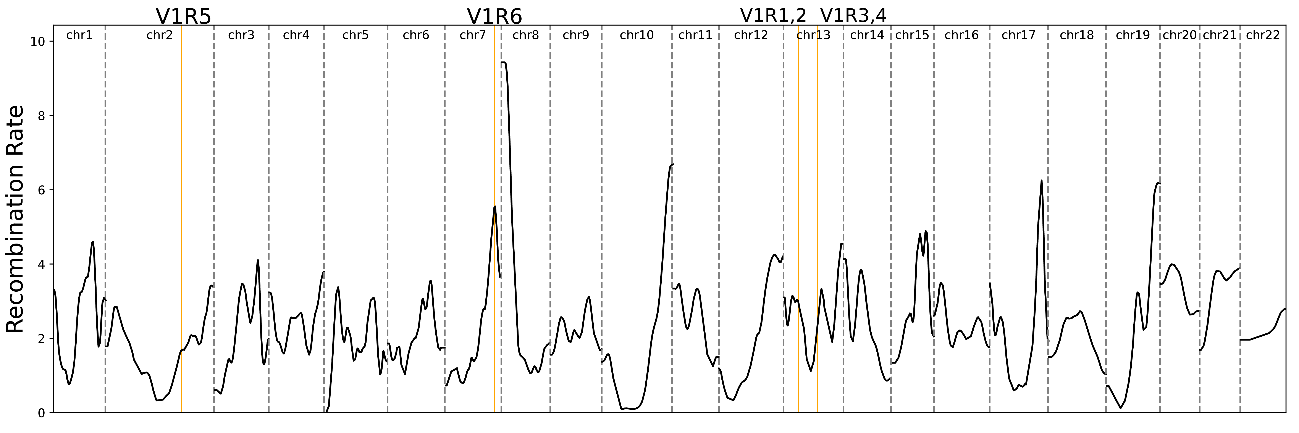


Figure S8 Recombination rate (cM/Mb) per chromosome in *Pundamilia nyererei* from the Lake Victoria lineage (Feulner et al., 2018). The positions of V1R genes are indicated by yellow lines.


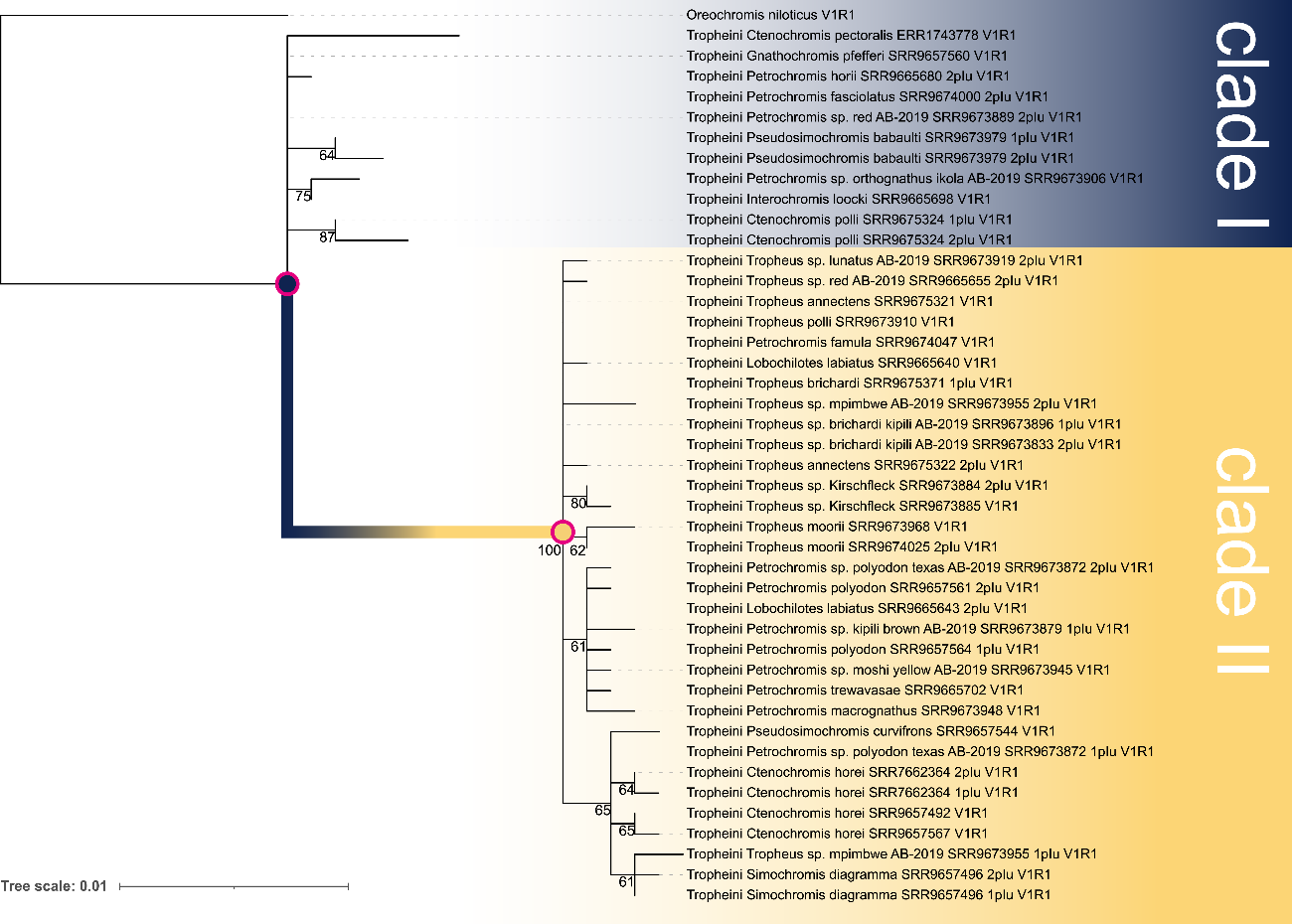


Figure S9 Phylogenetic tree of the V1R1 gene in Tropheini. Bootstrap values are shown only for nodes with support values of ≥60. The sequences are divided into two highly divergent alleles (Clades I and II). Recombination-derived sequences have been excluded.


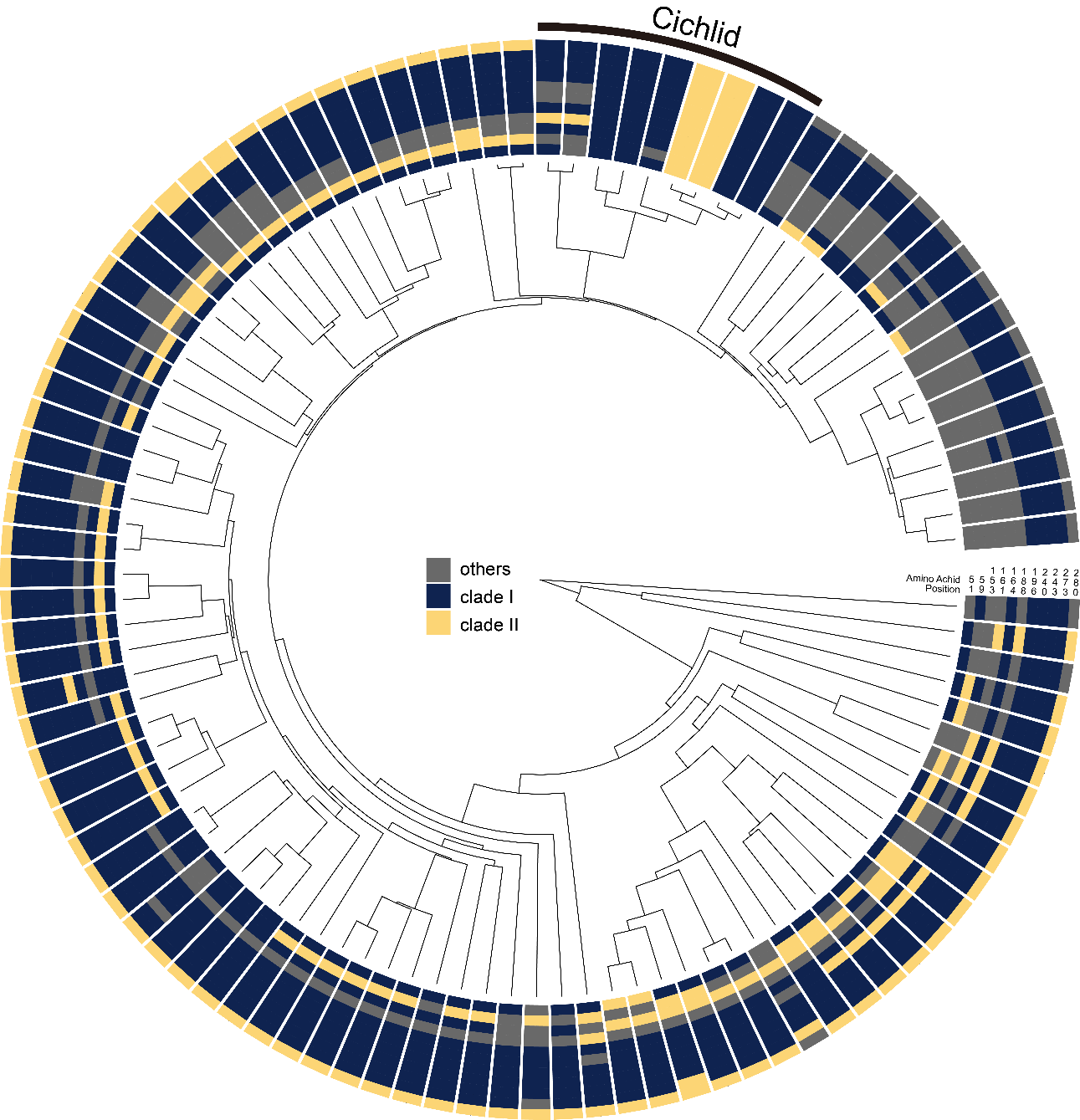


Figure S10 V1R1 alignment for 102 ray-finned fish species. The alignment displays sites corresponding to the two alleles (Clades I and II). Sites matching Clade I are highlighted in dark blue, those matching Clade II are highlighted in yellow, and all other sites are shown in gray.


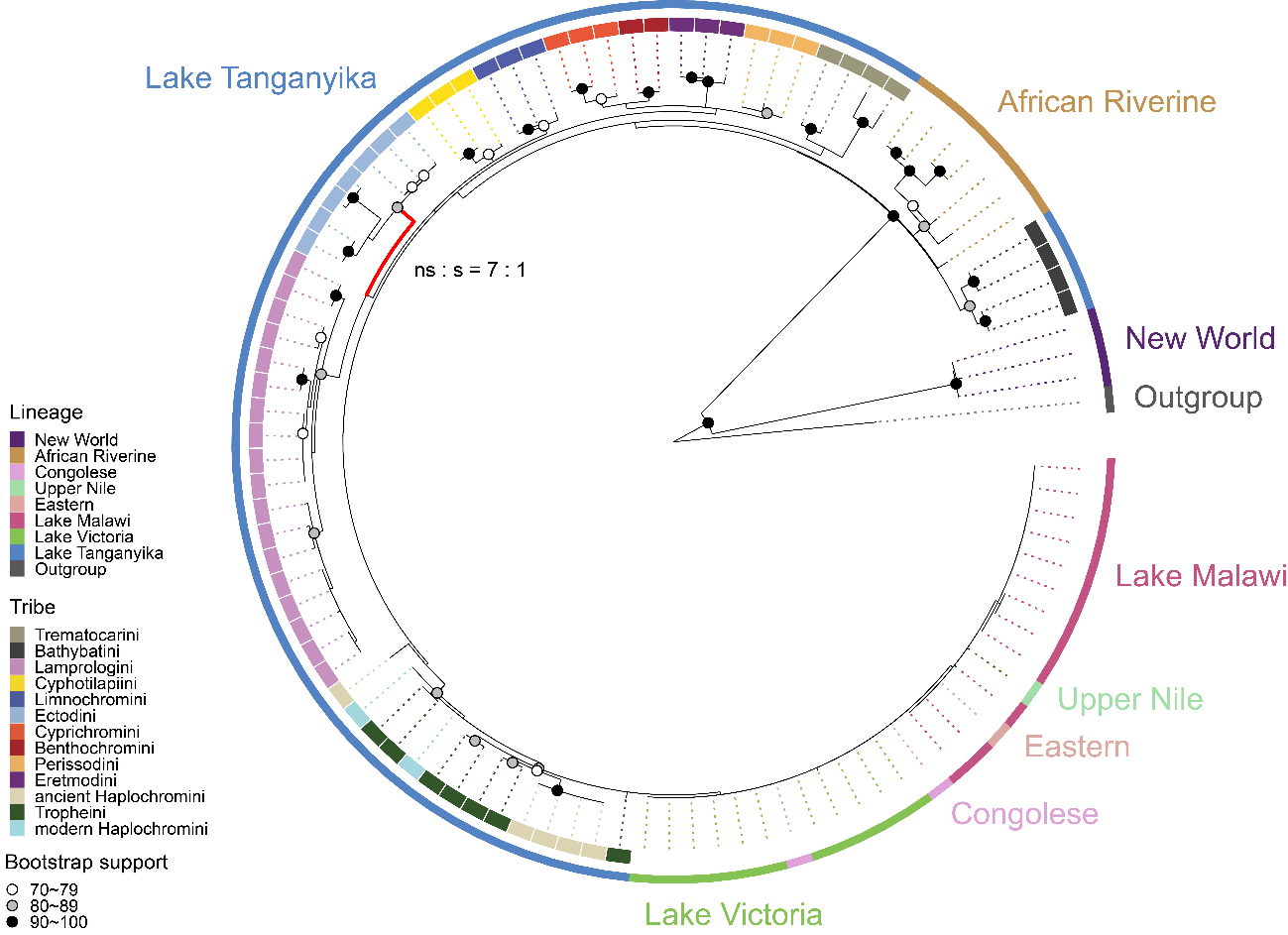


Figure S11 Phylogenetic tree of the V1R3 gene in cichlids. Nodes are marked with dots based on bootstrap values: black for values of ≥90, gray for 80–89, and white for 70–79. The inner circle represents tribes, whereas the outer circle indicates habitats. Branches under positive selection are highlighted in red (p = 0.0361, see Table S2). The number of nonsynonymous and synonymous substitutions for the branch is indicated as ns:s.


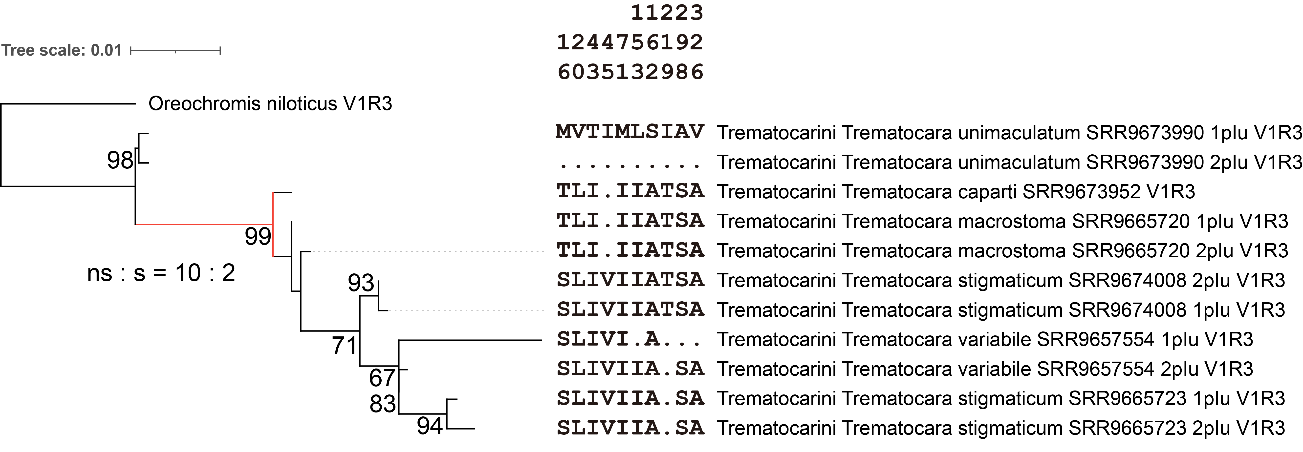


Figure S12 Phylogenetic tree and sequence alignment of the V1R3 gene in Trematocarini. Bootstrap values are shown only for nodes with support values of ≥60. *Trematocara unimaculatum*, which retains the ancestral sequence of V1R3, differs significantly from those of other species. The amino acid alignment displays only the sites with variations observed within Trematocarini. Dots indicate identity with the top sequence. Positive selection was not detected in the red branch (p = 0.0503, see Table S2). The number of nonsynonymous and synonymous substitutions in the red branch is indicated as ns:s.


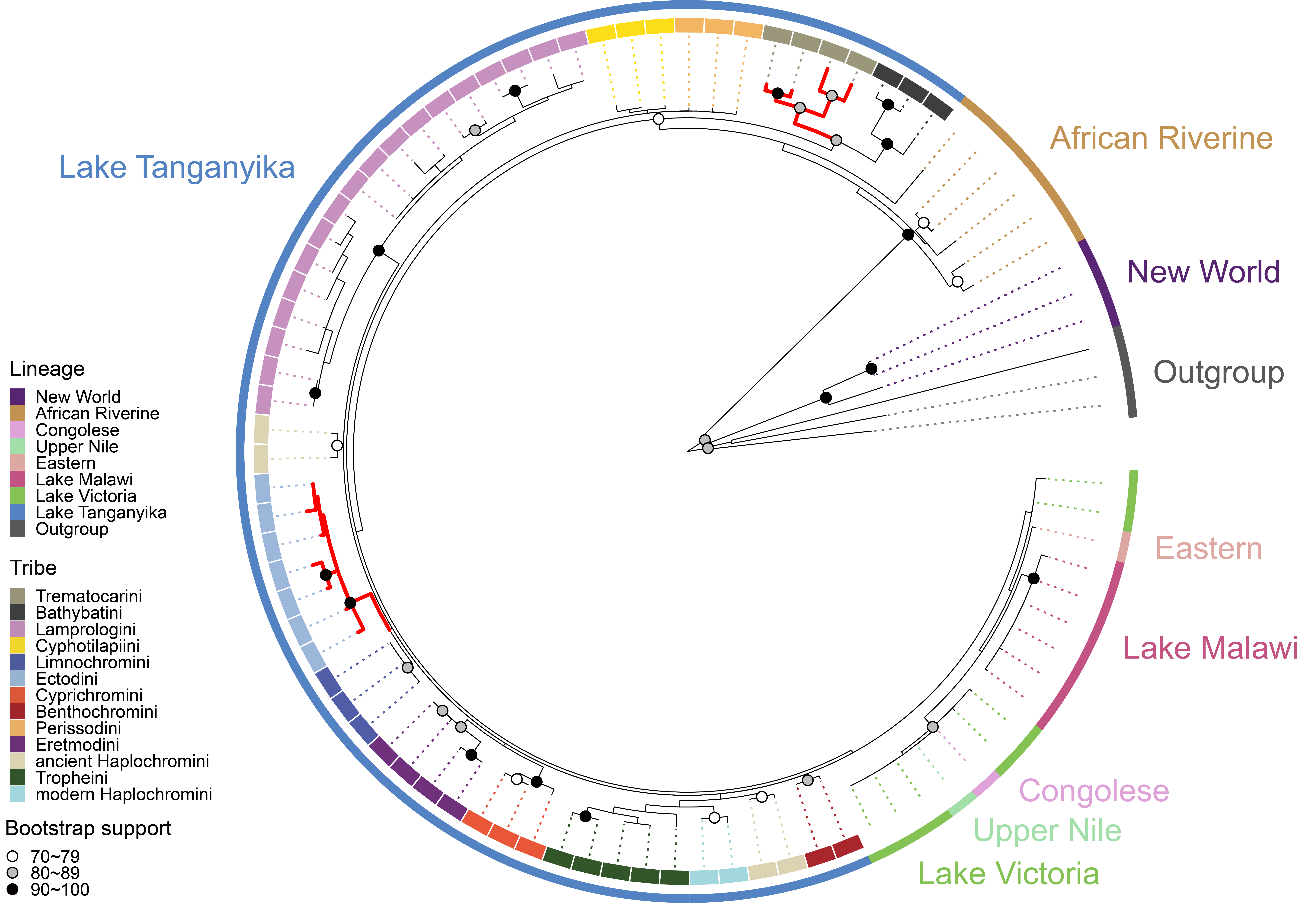


Figure S13 Phylogenetic tree of the V1R6 gene in cichlids. Nodes are marked with dots based on bootstrap values: black for values of ≥90, gray for 80–89, and white for 70–79. The inner circle represents tribes, whereas the outer circle indicates habitats. Branches under positive selection are highlighted in red (see Table S2).


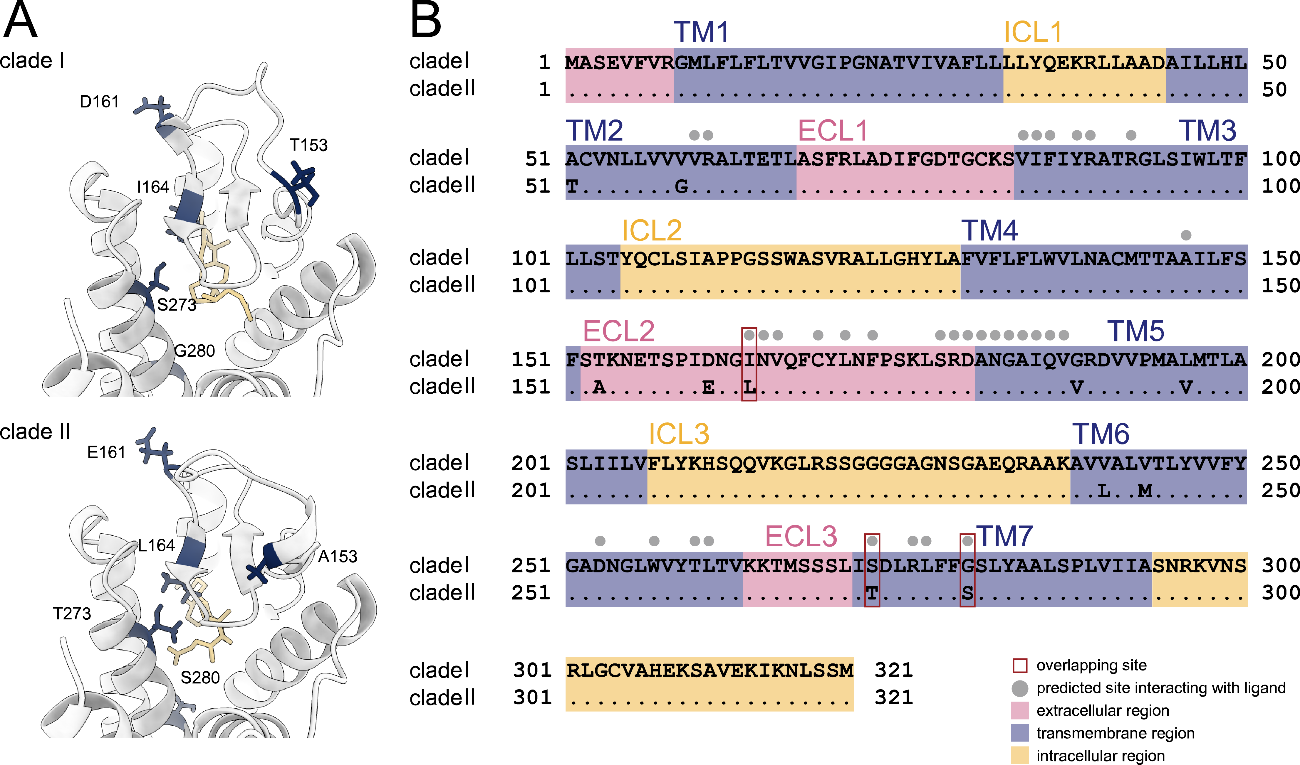


Figure S14 3D structure of cichlid V1R1. (A) 3D structure of V1R1 viewed from the extracellular side. Amino acids that differ between Clades I and II are shown in dark blue. The ligand used for analysis (lithocholic acid) is shown in yellow. (B) Predicted transmembrane regions of cichlid V1R1. The transmembrane regions were predicted using the Clade I sequence. Dots indicate amino acids identical to those in Clade I. The extracellular regions are shown in pink, transmembrane regions in blue, and intracellular regions in yellow. TM: transmembrane; ECL: extracellular loop; ICL: intracellular loop. Gray dots indicate the predicted ligand-binding sites. Red squares indicate ligand-binding sites that are also sites of substitution between alleles. (Fisher’s exact test: p = 0.0922)


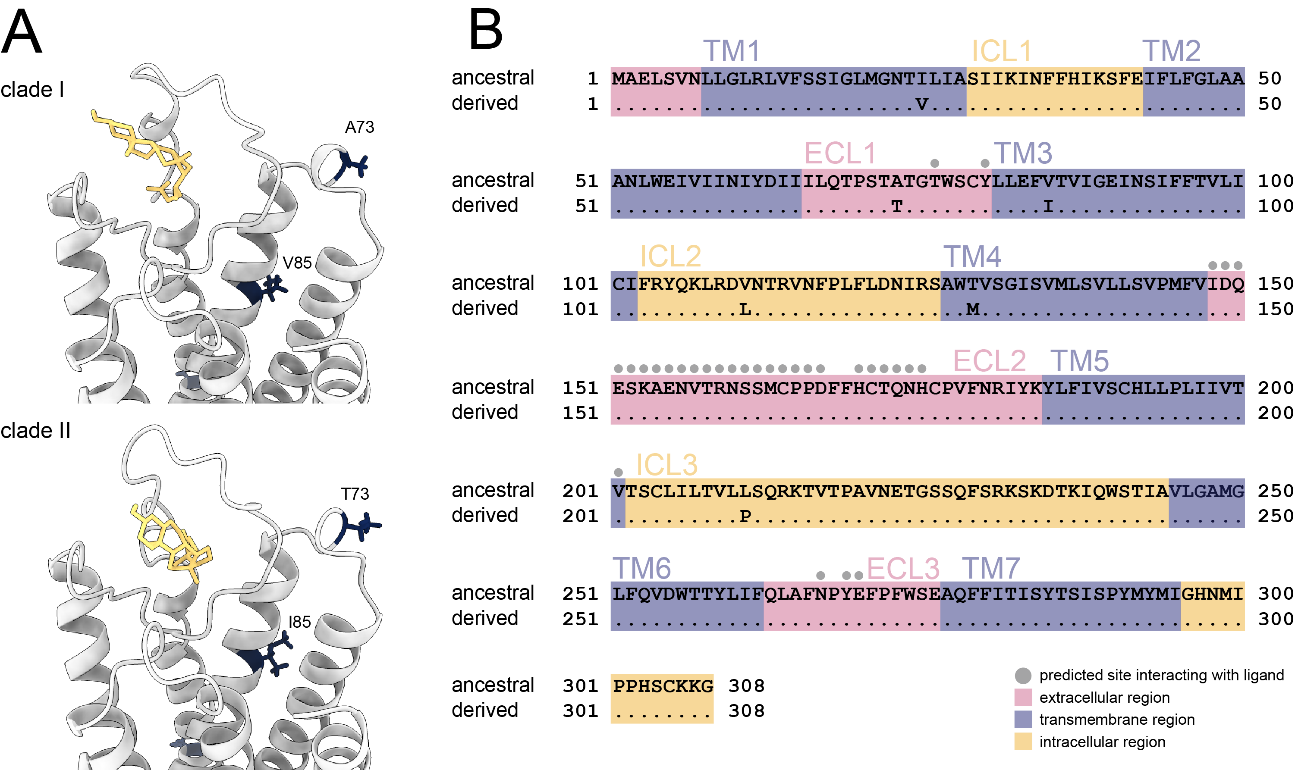


Figure S15 3D structure of cichlid V1R6. (A) 3D structure of V1R6 viewed from the extracellular side. Amino acids that differ between ancestral and derived alleles are shown in dark blue. The ligand used for analysis (lithocholic acid) is shown in yellow. (B) Predicted transmembrane regions of cichlid V1R6. The transmembrane regions were predicted using the ancestral allele sequence. Dots indicate amino acids identical to those in the ancestral allele. The extracellular regions are shown in pink, transmembrane regions in blue, and intracellular regions in yellow. TM: transmembrane; ECL: extracellular loop; ICL: intracellular loop. Gray dots indicate the predicted ligand-binding sites.


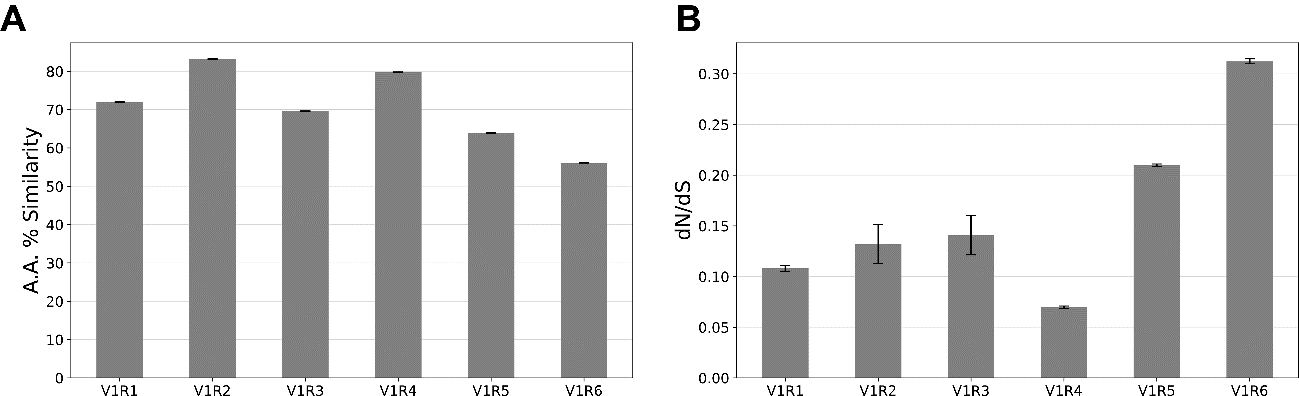


Figure S16 Sequence similarity and dN/dS ratios of V1R genes among 102 ray-finned fish species (A) Amino acid similarity of V1R genes. The average sequence similarity was calculated for all pairwise combinations among the 102 species. Error bars represent the standard errors. (B) dN/dS ratios of V1R genes. The average dN/dS ratio was calculated for all pairwise combinations among the 102 species. Error bars represent the standard errors.

Table S1 Short-read data from 28 tribes, 528 species, and 907 samples of cichlids registered in the NCBI Sequence Read Archive (SRA).

Table S2 Reference genomes used for mapping. Because the NeoBri1.0 genome has not been assembled to the chromosome level, we performed genome alignment using RagTag (Alonge et al., 2022) to scaffold NeoBri1.0 against O_niloticus_UMD_NMBU. Furthermore, repetitive elements in all reference genomes were masked by replacing them with N using RepeatMasker (Smit, AFA, Hubley, R & Green, P. RepeatMasker Open-4.0. 2013–2015; http://www.repeatmasker.org).

Table S3 Selection analyses and likelihood ratio test results.

**Reference**

Alonge, M., Lebeigle, L., Kirsche, M., Jenike, K., Ou, S., Aganezov, S., Wang, X., Lippman, Z. B., Schatz, M. C., & Soyk, S. (2022). Automated assembly scaffolding using RagTag elevates a new tomato system for high-throughput genome editing. *Genome Biology*, *23*(1), 258. https://doi.org/10.1186/s13059-022-02823-7

Feulner, P. G. D., Schwarzer, J., Haesler, M. P., Meier, J. I., & Seehausen, O. (2018). A Dense Linkage Map of Lake Victoria Cichlids Improved the Pundamilia Genome Assembly and Revealed a Major QTL for Sex-Determination. *G3* , *8*(7), 2411–2420. https://doi.org/10.1534/g3.118.200207

Matschiner, M., Böhne, A., Ronco, F., & Salzburger, W. (2020). The genomic timeline of cichlid fish diversification across continents. *Nature Communications*, *11*(1), 5895. https://doi.org/10.1038/s41467-020-17827-9

Meier, J. I., Marques, D. A., Mwaiko, S., Wagner, C. E., Excoffier, L., & Seehausen, O. (2017). Ancient hybridization fuels rapid cichlid fish adaptive radiations. *Nature Communications*, *8*, 14363. https://doi.org/10.1038/ncomms14363

Nakamura, H., Aibara, M., & Nikaido, M. (2023). Ancient standing genetic variation facilitated the adaptive radiation of Lake Victoria cichlids. *Genes & Genetic Systems*. https://doi.org/10.1266/ggs.23-00024

Ronco, F., Matschiner, M., Böhne, A., Boila, A., Büscher, H. H., El Taher, A., Indermaur, A., Malinsky, M., Ricci, V., Kahmen, A., Jentoft, S., & Salzburger, W. (2021). Drivers and dynamics of a massive adaptive radiation in cichlid fishes. *Nature*, *589*(7840), 76–81. https://doi.org/10.1038/s41586-020-2930-4
